# Utility of autologous fecal microbiota transplantation and elucidation of microbiota in diversion colitis

**DOI:** 10.1002/deo2.63

**Published:** 2021-10-31

**Authors:** Kentaro Tominaga, Atsunori Tsuchiya, Takeshi Mizusawa, Asami Matsumoto, Ayaka Minemura, Kentaro Oka, Motomichi Takahashi, Tomoaki Yoshida, Yuichi Kojima, Kohei Ogawa, Yuzo Kawata, Nao Nakajima, Naruhiro Kimura, Hiroyuki Abe, Toru Setsu, Kazuya Takahashi, Hiroki Sato, Satoshi Ikarashi, Kazunao Hayashi, Ken‐ichi Mizuno, Junji Yokoyama, Yosuke Tajima, Masato Nakano, Yoshifumi Shimada, Hitoshi Kameyama, Toshifumi Wakai, Shuji Terai

**Affiliations:** ^1^ Division of Gastroenterology and Hepatology Graduate School of Medical and Dental Sciences Niigata University Niigata Japan; ^2^ R&D Division Miyarisan Pharmaceutical Co. Ltd. Saitama Japan; ^3^ Division of Digestive and General Surgery Graduate School of Medical and Dental Sciences Niigata University Niigata Japan

**Keywords:** colostomy, diversion colitis, *Enterobacteriaceae*, fecal microbiota transplantation, intestinal microbiota

## Abstract

**Objectives:**

Diversion colitis (DC) is an inflammatory disorder caused by interruption of the fecal stream and subsequent nutrient deficiency from luminal bacteria. The utility of fecal microbiota transplantation (FMT) for DC was recently investigated; however, the precise pathogenesis of this condition remains unclear. This study aimed to evaluate the utility of autologous FMT in DC and to determine the related changes in the intestinal microbiota.

**Methods:**

Autologous FMT was performed to reestablish the intestinal microbiota in five patients (average age, 64.6 ± 8.3 years) with DC. They underwent double‐ended colostomy. We assessed the diverted colon by endoscopy and evaluated the microbiota before and after FMT using the 16S rRNA gene sequencing method.

**Results:**

All five patients had mild inflammation (ulcerative colitis endoscopic index of severity [UCEIS] 2–3) in the diverted colon based on the colonoscopic findings. Three patients presented with symptoms, such as tenesmus, mucoid stool, and bloody stool. With FMT treatment, all patients achieved endoscopic remission (UCEIS score of 0 or 1) and symptomatic improvement. We observed a significantly decreased α‐diversity in DC patients compared to healthy controls. The frequency of aerobic bacteria, such as *Enterobacteriaceae*, in the diverted colon decreased after autologous FMT.

**Conclusions:**

This study was the first to show that the microbiota in the diverted colon was significantly affected by autologous FMT. Since interruption of the fecal stream is central to the development of DC, FMT can be considered a promising treatment.

## INTRODUCTION

Diversion colitis (DC) was first described by Morson and Dawson in 1934 as nonspecific inflammation of the diverted colon.^1^ Glotzer et al. termed this inflammation “DC” in 1981.^2^ A prospective study showed that colitis was detected via endoscopic studies on patients who underwent colostomy 3–36 months prior.^3^ Approximately one third of DC patients had symptoms, such as abdominal discomfort, tenesmus, mucoid discharge, and rectal bleeding.^4^, ^5^ The change in the microbiota of the diverted colon was reportedly a major cause of DC^6^; however, the precise pathogenesis of this condition remains unclear. Gundling et al. first reported a successful autologous fecal transplantation in a patient with chronic DC.^7^ We have previously reported a case with clinical findings of DC and microbiota in a diverted ileal pouch before and after autologous fecal microbiota transplantation (FMT).^8^ We also previously reported on the microbiota, intestinal short‐chain fatty acids, and immunoglobulin A (IgA) in the diverted colon in eight patients who underwent colostomies.^9^ In the present study, five of these eight patients underwent autologous FMT, and we compared the endoscopic findings and intestinal microbiota before and after the treatment. This present study aimed to evaluate the utility of autologous FMT and to determine the changes in the intestinal microbiota of DCs.

## METHODS

### Subjects and study protocol

**FIGURE 1:**
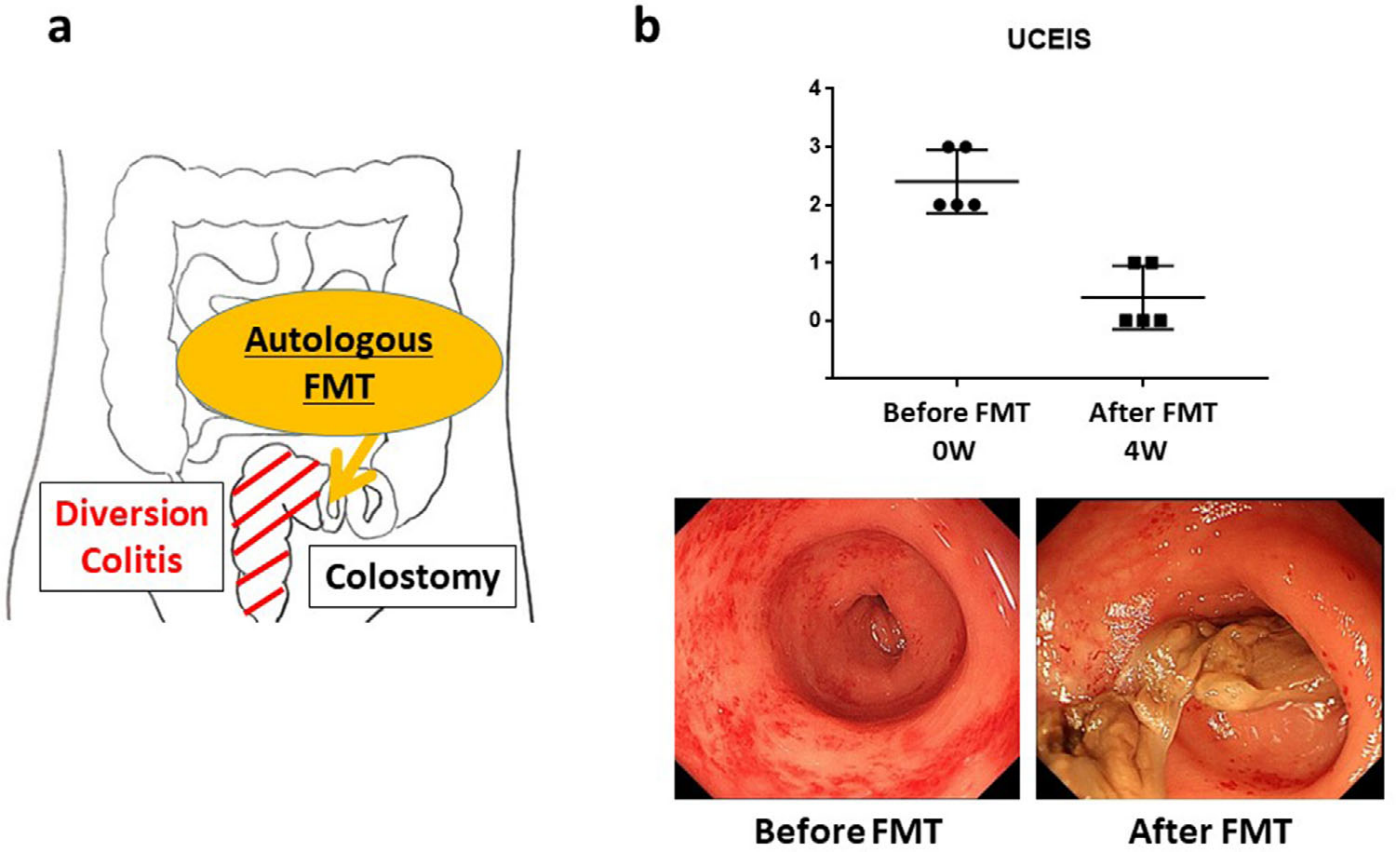
(a) The schema of autologous fecal microbiota transplantation (FMT) for diversion colitis. (b) Ulcerative colitis endoscopic index of severity (UCEIS); endoscopic findings of the diverted colon before and after autologous FMT

Written informed consent was obtained from the patients for the publication of the report and accompanying images. We also obtained written informed consent from the patients to receive auto FMT and provide stool samples. The study (including the informed consent for collecting fecal samples from healthy individuals) was reviewed and approved by the Institutional Review Board of Niigata University (2017‐0154). The subjects were five patients (64.6 ± 8.3 years) (male:female = 0:5) who underwent colostomy within the previous 6–40 months. Two of these patients had a history of rectal cancer, while the remaining three patients had a retroperitoneal abscess, ovarian cancer, and rectovaginal septum cancer, respectively (Table 1). We assessed the diverted colon endoscopically and evaluated its severity using the ulcerative colitis endoscopic index of severity (UCEIS). None of the patients received antibiotics or probiotics for 1 month leading up to the date of colonoscopy. Furthermore, none of the patients had previously undergone enema with short‐chain fatty acids, mesalazine, or corticosteroids for DC. We performed autologous FMT to reestablish the intestinal microbiota for these patients. We injected 10–20 ml of saline‐diluted autologous feces from the stoma to the diverted colon using a Nelaton catheter. Nine FMTs were performed for over 4 weeks (when changing the stoma pouch once every 3 days) (Figure 1a).

**TABLE 1:** Patient characteristics

Case (number)	Age (years)	Sex	Diagnosis	Operative procedure	Symptom	UCEIS	Postoperative period
1	49	F	Rectal cancer	Low anterior resection + colostomy	None	2	6M
2	66	F	Rectal cancer	Low anterior resection + colostomy	None	2	6M
3	67	F	Retroperitoneal abscess	Left hemicolectomy + transverse colostomy	Mucous stool	2	16M
4	74	F	Ovarian cancer	Transverse colostomy	Mucous stool	3	18M
5	67	F	Rectovaginal septum cancer	Transverse colostomy	Bloody stool and tenesmus	3	40M

Abbreviation: UCEIS, ulcerative colitis endoscopic index of severity.

We assessed the intestinal microbiota of the diverted colon using a next‐generation sequencer (Illumina MiSeq; Illumina, San Diego, CA, USA). We conducted a 16S rRNA gene sequence analysis. The composition of the intestinal microbiota was evaluated using quantitative insights on microbial ecology. To evaluate diversity, feces from six healthy individuals (38.5 ± 3.4 years) (male:female = 4:2) were used as controls. Data on the intestinal microbiota in the diverted colon before and after FMT treatment were analyzed using the linear discriminant analysis (LDA) effect size.

### Intestinal sample collection

We collected the intestinal samples from the diverted colon (the rectum) using net forceps endoscopically without a biopsy. Intestinal samples (approximately 100 mg) were suspended in 900 μl of guanidine thiocyanate solution (100 mM Tris–HCl [pH 9.0], 40 mM EDTA, and 4 M guanidine thiocyanate) and frozen at −80°C until further analysis.^10^

### DNA preparations from intestinal samples

The collected samples were sent to the laboratory of Miyarisan Pharmaceutical Co. Ltd. and stored at 4°C. DNA was extracted from collected intestinal samples using a bead beating method and purified, according to a previously reported method.^11^ The amount of DNA was determined using the Quanti Fluor ONEdsDNA System and Quantus Fluorometer (Promega, Madison, WI, USA).

### PCR amplification and analysis of 16S rRNA gene sequence

The V3‐V4 region of the 16S rRNA gene was polymerase chain reaction (PCR) amplified from stool DNA samples using a TaKaRa Ex Taq Hot Start PCR mixture (Takara Bio, Shiga, Japan). The primers used for PCR amplification were 341F and 785R, which contained the Illumina index and sequencing adapter overhangs.^12^ PCR assays were performed using a TaKaRa PCR Thermal Cycler Dice Touch device (Takara Bio) with the following parameters: initial denaturation at 98°C for 30 s, followed by 35 cycles of 98°C for 10 s, and 60°C for 30 s, with a final extension step at 72°C for 5 min. The PCR products were purified, and size was selected using SPRIselect (Beckman Coulter, Brea, CA, USA). DNA concentrations were quantified with a QuantiFluor ONEdsDNA System and Quantus Fluorometer (Promega), and equal amounts of purified PCR products were pooled for subsequent Illumina MiSeq sequencing. Sequencing was carried out with a Miseq Regent Kit V3 (600 cycles) (Illumina), according to the manufacturer's instructions. Sequence processing and quality assessment were performed using the QIIME package (version 1.9.1) (http://qiime.org), open‐source software created to address the problem of obtaining sequencing data from raw sequences for interpretation and database deposition.^13^ To obtain an overall diversity analysis for subsequent comparative and statistical evaluations, we merged the biological observation matrix (BIOM) tables provided by QIIME into a unique BIOM table using a script included in the QIIME package. Paired‐end reads were merged using the Fastq‐join script in ea‐utils with the parameters *m* = 6 and *p* = 20 and then quality‐filtered using QIIME's script split_libraries_fastq.py (*r* = 3, *p* = 0.75, *q* = 20, *n* = 0). De novo and reference‐based chimera detection and removal were performed using USEARCH v6.1 with the Greengenes v13.8 database. Operational taxonomic units (OTUs) were chosen using an open reference OTU‐picking pipeline against the 97% identity of the pre‐clustered Greengenes v13.8 database using UCLUST. According to the manufacturer, the QIIME alpha diversity analysis script is used to perform rarefaction analysis by subsampling the OTU biom table on the basis of the minimum rarefaction depth value chosen by the user depending on the minimum number of sequences/samples obtained. For our subset, this value was 16087. Then, using different metrics, alpha diversity was computed for each rarefied OTU table. We used two non‐phylogeny‐based metrics: Chao 1 and the Observed_OTUs. After performing the rarefaction evaluation, the QIIME beta diversity analysis script was used to compute beta diversity with the rarefied OTUs table using different metrics. We used phylogeny‐based metrics (unweighted and weighted UniFrac).^14^ Finally, the script was used to obtain a distance metric to compute the principal coordinate analysis (PCoA) and convert it into plots for the visualization of results. We used permutational multivariate analysis of variance (PERMANOVA) to evaluate the statistical significance of beta diversity distances.

### Statistical analysis

The alpha‐diversity indices were compared between the groups using the Mann–Whitney *U* test in R (The R Foundation for Statistical Computing, Vienna, Austria). The significance of beta‐diversity was evaluated by PERMNOVA in QIIME 1.9.1. The enriched bacteria in each group were identified by LDA effect size (LEfSe)^15^; LDA values >3 were considered significant.

## RESULTS

### Endoscopic evaluation and symptoms

Five patients underwent stoma surgery within 6–40 months prior to this study. Since the method for assessing inflammation of DC has not yet been established, we performed endoscopy for all patients and evaluated the inflammation of the diverted colon using the UCEIS score.^9^ All five patients had mild inflammation (UCEIS score 2 or 3), such as erythema and mucosal friability, as detected via endoscopy (Table 1). We could detect mucosal bleeding and blurring of vascular pattern in all five patients and erosions in two patients. With autologous FMT treatment, all patients achieved endoscopic remission (UCEIS score 0 or 1) at 1 month after the treatment (Figure 1b).

Three of the five patients showed symptoms, such as tenesmus, mucus, or bloody stools (Table 1). Two patients had no obvious symptoms at the time of endoscopy, but sometimes had tenesmus and mucous stools during the 6 months after surgery. Taking the inflammatory findings of the endoscopy into consideration, FMT treatment was performed with the patient's consent to prevent symptom recurrence. With autologous FMT treatment, all patients achieved symptomatic improvement within 2 weeks of starting FMT. No adverse effects of autologous FMT were observed.

### Intestinal microbiota

**FIGURE 2:**
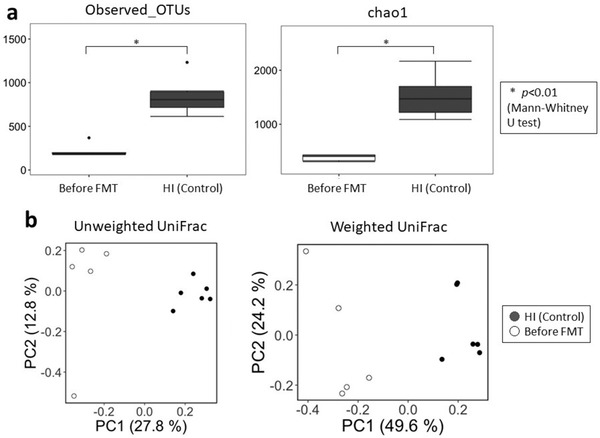
(a) The α‐diversity data of this study. Comparison of diversion colitis (DC) patients before fecal microbiota transplantation (FMT) and healthy individuals (HI). Bars show the standard deviation of the data. Data were analyzed using the Mann–Whitney *U* test (*p < 0.01). (b) The β‐diversity data of this study. Comparison of DC patients before FMT and healthy individuals (HI). Data were analyzed using the UniFrac distance analysis. Data analyzed using the PERMANOVA and both the weighted and unweighted UniFrac showed significant differences between the two groups (*p* < 0.01)

We analyzed the intestinal microbiota using 16S rRNA gene sequencing and detected significantly decreased α‐diversity (Observed OTUs and chao1, *p* < 0.01; the data were analyzed using the Mann–Whitney *U* test) in DC patients compared to healthy controls (Figure 2a). These results indicate that the intestinal microbiota of DC patients is less diverse than that of young healthy individuals (control). Regarding the β‐diversity, clusters were formed in DC patients and healthy controls (Figure 2b). The β‐diversity also showed a significant difference between DC patients and healthy controls (weighted and unweighted UniFrac, *p* < 0.01; the data were analyzed using the PERMANOVA), which indicated a low similarity of microbiota between the two groups.

**FIGURE 3:**
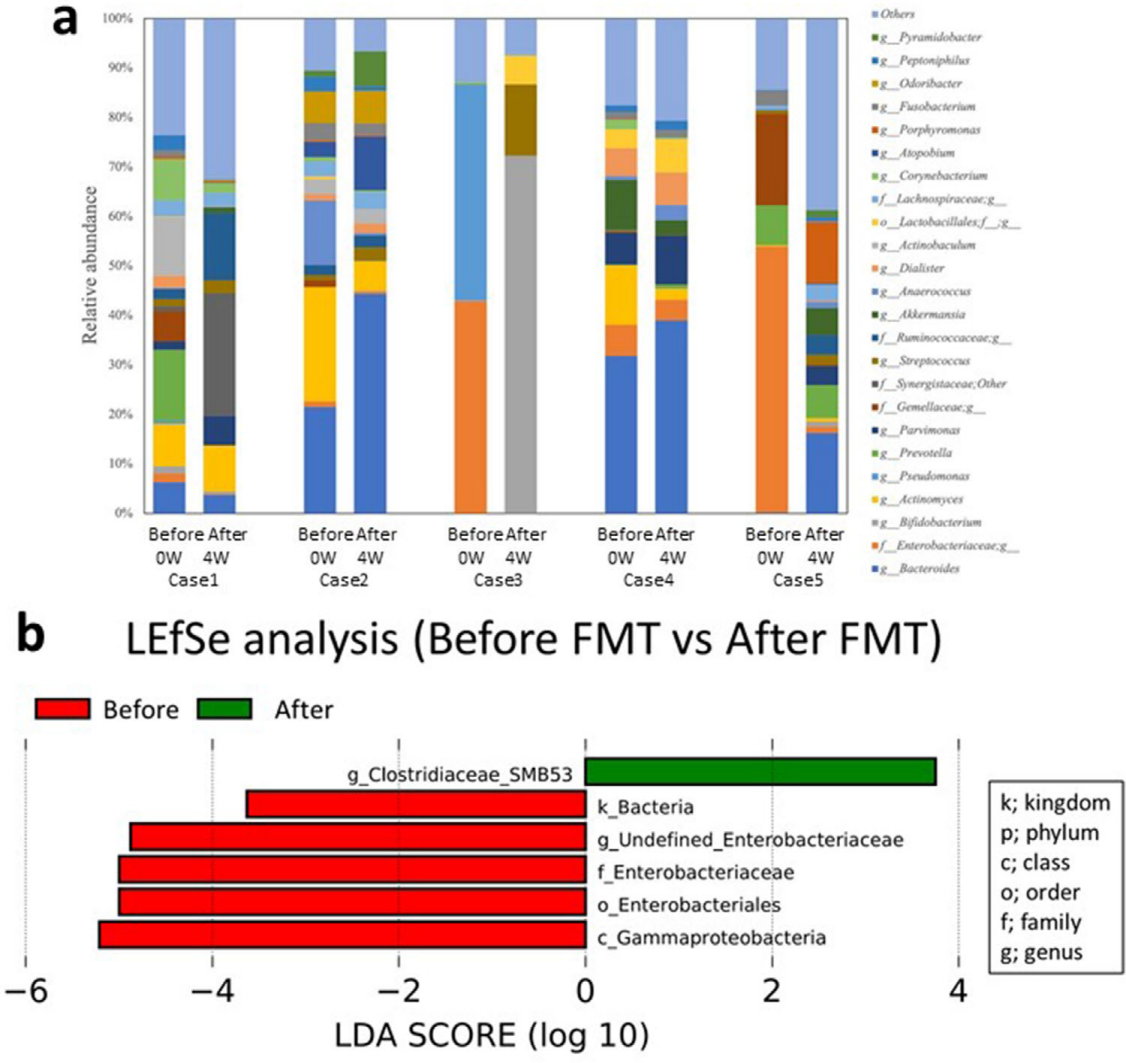
(a) Analysis of the intestinal microbiota at the genus level in the diverted colon before and after FMT treatment. (b) Analysis of the intestinal microbiota in the diverted colon before and after fecal microbiota transplantation (FMT) treatment. Data were analyzed using the linear discriminant analysis effect size. Red signifies the presence of more microbiota before the treatment, and green signifies the presence of more microbiota after the treatment

We analyzed the intestinal microbiota in the diverted colon before and after FMT treatment using the LDA effect size. The analysis of the intestinal microbiota at the genus level showed a significantly decreased frequency of *Enterobacteriaceae* in the diverted colon after FMT treatment compared to before FMT (Figure 3a). After the treatment, in addition to the significantly decreased frequency of *Enterobacteriaceae* at the genus level, the frequency of *Enterobacteriaceae* at the family level, *Enterobacteriales* at the order level, and *Gamma‐proteobacteria* at the class level were also significantly decreased (Figure 3b). Moreover, there was a significantly increased frequency of *Clostridiaceae* at the genus level after treatment (Figure 3b).

## DISCUSSION

The prospective study reported that almost all cases exhibited colitis, based on the endoscopic findings, at 3–36 months after the colostomy.^3^ These findings include erythema, diffuse granularity, and blurring of vascular pattern in approximately 90% of the population. It is also associated with mucosal friability (80%), edema (60%), aphthous ulceration, and bleeding, to varying degrees.^2^, ^16^, ^17^ The UCEIS score is often used to evaluate endoscopic findings in DC. In our study, mild colitis (UCEIS score 2 or 3) was observed in all patients endoscopically at 6–40 months after the colostomy.

Re‐anastomosis was proven to be consistent and effective for DC. Non‐surgical treatments, including antibiotics, glucocorticoids, short‐chain fatty acid enemas, and 5‐aminosalicylic acid, are viable alternatives.^6^ Gundling et al. first reported that autologous FMT was an effective and safe option for relapsing DCs after the standard therapies have failed.^7^ Few cases have reported the utility of autologous FMT for DC. In this study, the method of administration was determined according to the state of inflammation with reference to our case.^8^ Unlike existing methods, an endoscope is not required, and there is no need to stay in bed for a long time after FMT treatment.^7^, ^18^ This study showed that administering a small amount of feces to the diverted colon during stoma replacement reduced inflammation and relieved symptoms. Autologous FMT was safe and inexpensive with a low risk of complications.

The interruption of the fecal stream is central to the development of DC. Baek et al. reported decreased anaerobes, such as *Lactobacillus* and *Bifidobacterium*, in DC.^19^ We have reported the microbiota in DCs. The microbiota showed a change in the proportion of some species, especially *Lactobacillus*, which was significantly decreased in the diverted colon.^9^ We also showed that intestinal short‐chain fatty acids were significantly decreased, and intestinal IgA was significantly increased in the diverted colon. Our results suggested that short‐chain fatty acids affected the microbiota, such as *Lactobacillus*, which plays a role in the immunity of the diverted colon.^9^ However, the changes in the intestinal microbiota after FMT remain unknown. In the present study, we observed a significantly decreased frequency of aerobic bacteria, such as *Enterobacteriaceae*, and a significantly increased frequency of anerobic bacteria, such as *Clostridiaceae*, after autologous FMT in the diverted colon. Neut et al. reported that the aerobic bacteria increased in the diverted colon and are a major cause of DC,^20^ which is similar to our findings.

A decreased diversity of intestinal microbiota has been noted in patients with inflammatory bowel diseases, such as ulcerative colitis or Crohn's disease.^21^, ^22^ However, there are no reports on the diversity of intestinal microbiota in patients with DC. In our study, we found a decrease in the α‐diversity of intestinal microbiota in patients with DC compared with healthy controls. We also showed a significant difference in β‐diversity between the two groups. These results indicate that the intestinal microbiota of DC patients forms different clusters and is less diverse than that of healthy controls. As a limitation, further research is needed due to the small number of cases and bias by age and gender. Follow‐up information would also be important, but since all five patients underwent stoma closure within 2 weeks after FMT treatment, subsequent long‐term follow‐up and endoscopic evaluation were not possible. We believe that further studies are necessary.

This was the first report to show significant changes in the intestinal microbiota before and after autologous FMT in DC patients. Since DC can be resolved by reestablishing the microbiota, autologous FMT is a viable treatment option.

In conclusion, autologous FMT resulted in a change in the microbiota, followed by improvement of symptoms and colonoscopy findings. Further studies are necessary to elucidate the mechanisms behind DC. Fecal stream interruption is central to DC development. Our study emphasized the significance of autologous FMT as a means of safely reestablishing the microbiota in DC.

## CONFLICT OF INTEREST

Miyarisan Pharmaceutical Co. Ltd. supported this study in the analysis of the microbiota.

## FUNDING INFORMATION

The present study has received financial support from Takeda Japan Medical Office Funded Research Grant 2018, Tsukada Medical Funded Research Grant, Grant‐in‐Aid for Young Scientific Research (19K17393) from the Ministry of Education, Science, Technology, Sports, and Miyarisan Pharmaceutical Co. Ltd.
